# Validity and Reliability of the Wii Balance Board for Static Balance Assessment in Healthy Young Adults

**DOI:** 10.21315/mjms2019.26.2.12

**Published:** 2019-04-30

**Authors:** Kitchana Kaewkaen, Suraphong Uthama, Worasak Ruengsirarak, Rungthip Puntumetakul

**Affiliations:** 1 Department of Physical Therapy, School of Health Science, Mae Fah Luang University, Chiang Rai, Thailand; 2 School of Information Technology, Mae Fah Luang University, Chiang Rai, Thailand; 3 Department of Physical Therapy, Faculty of Associated Medical Science, Khon Kaen University, Thailand; 4 Research center in Back, Neck, Other Joint Pain and Human Performance (BNOJPH) Khon Kaen University, Thailand

**Keywords:** balance assessment, young adults, Wii Balance Board

## Abstract

**Introduction:**

The Wii Balance Board (WBB) is a commonly used tool for balance assessment, however the inconsistency in the reported validity for the WBB when used for the assessment of healthy young adults needs to be clarified.

**Aim:**

To investigate the concurrent validity and reliability of the WBB for balance assessment in healthy young adults.

**Methods:**

Thirty-two young adults participated in this study. Their ability to balance was tested while standing on a WBB and a laboratory-grade force platform, under three conditions: feet together with eyes open, feet together with eyes closed and semi-tandem standing with eyes open. They had 10 min resting period between tests. The agreement between the WBB and the laboratory-grade force platform was investigated, and the reliability of the WBB was determined.

**Results:**

A poor agreement between the WBB and the laboratory-grade force platform was found for all standing conditions [intraclass correlation coefficient (ICC) = 0.03 to 0.07]. A moderate to high reliability was found for the WBB for balance assessment in healthy young adults (ICC = 0.66 to 0.76).

**Conclusion:**

The WBB was found to be a reliable tool for static balance assessment in healthy young adults. However, it had poor validity compared to the laboratory-grade force platform.

## Introduction

Balance control plays an important role in the performance of daily life activities and contributes to working effectively. The upright position in standing or walking requires control of the centre of gravity within the base of support. Several instruments have been used for balance assessment, for both static and dynamic balance, but it is the laboratory-grade force platform that is considered to be the gold standard tool for static balance assessment (1). The use of laboratory-grade force platforms is restricted, however, as they are both expensive and not very portable outside of the balance clinic. The Wii Balance Board (WBB) is, on the other hand, a viable low-cost alternative device for balance assessment and has many advantages over laboratory-grade force platforms.

WBBs are increasingly used in both clinical assessment and interventions, and for research (2, 3). Several studies report a range of findings for the psychometric properties of the WBB. Positive results show the WBB to have a high degree of validity when compared with a standard laboratory-grade force platform (4, 5). This positive psychometric finding is also supported by a good-to-excellent reliability rating. A less positive psychometric finding includes the wide range of correlations reported for the WBB compared to a standard laboratory-grade force platform (6, 7, 8). These range from non-significant to a correlation of *r* = 0.497. The latter result came from a comparison between the WBB and the Neurocom Smart Balance Master System reported by Wright et al. (6), who studied healthy young adults and participants with mild traumatic brain injuries. A low reliability was also found for the WBB in young adults (7).

More evidence is needed to clarify the psychometric properties of the WBB and to provide an insight into the contradictory results concerning balance assessment in young adults from previous studies. Clark et al. (8) suggested that test duration (less than 30 s) and low sample size are, potentially, the influencing factors in the poor validity reported in previous studies. The purpose of this study, therefore, is to address these factors and to clarify the psychometric properties of the WBB as a clinical tool. More specifically, the aim of this study is to investigate the concurrent validity and reliability of the WBB for balance assessment in healthy young adults.

## Methods

### Study Design and Setting

A cross-sectional study was conducted at the Human Interface and Mobile Devices Laboratory, Faculty of Information Technology, Mae Fah Luang University (MFU), Thailand.

### Participants

The effect size was hypothesised at 0.9 [intraclass correlation coefficient (ICC) for the alternative hypothesis] while the ICC for the null hypothesis was assumed to be 0.7. A minimum sample size of 25 participants is required to measure the sway path twice for each participant, for both the WBB and the laboratory-grade force platform. This is done in order to achieve statistical significance for an alpha-value set at 0.05 and with the minimum power of at least 90% (9).

A total of 32 participants took part in this study. All the participants recruited in this study satisfied the inclusion criteria: healthy young adults, aged between 18–40 years, able to stand independently for at least 30 s. Participants were excluded if they had a past medical history which could potentially affect their ability to balance, such as Parkinson’s disease or a stroke. Those with a dynamic balance impairment (as tested by the Timed Up and Go test), lower extremity weakness (as tested by the Five Times Sit-To-Stand test) or an injury in a lower extremity were also excluded.

All participants understood the study protocols as explained by a research assistant, and they all signed an informed consent form before participating in the study. The research protocol was approved by the Mae Fah Luang University Ethics Committee, according to the Declaration of Helsinki. The ethical record number is REH-60094.

### Procedure

**Figure 1:**
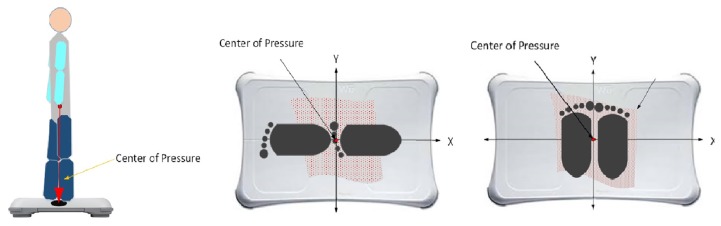
Foot position for standing balance testing

After screening for eligibility, the static balance ability of the participants was assessed by the same research assistant. They participants were asked to stand on the WBB (Nintendo^TM^, Tokyo, Japan) and then on a laboratory-grade force platform (Zebris Medical GmbH, Germany), with a safety belt, for three conditions (Figure 1):

Condition 1: Participants placed their feet close together on either side of the centreline of the WBB with their eyes open, and were instructed to stand as still as possible, keeping their arms to the sides of their body, and to focus on a point on the wall directly in front of them. A previous study has reported the positive effect of gaze stabilisation on postural stability in young adults (10).
Condition 2: As in Condition 1, participants placed their feet close together on either side of the centreline of the WBB, but this time with their eyes closed.
Condition 3: Participants were instructed to stand as in Condition 1, but with their feet in a semi-tandem standing position, placed ‘one in front of the other’, with the dominant foot placed behind the non-dominant foot on the long centreline of the WBB, with their eyes open.

Participants were requested to stand for 30 s for each test, and performed the test twice for each condition, with a 10-min rest between tests (7). They were allowed one practice test for each condition to familiarise themselves with the instructions and the test requirements. This was repeated for both measurement platforms.

On completion of the testing, the averages for each participant for each condition and for each platform were used for statistical analysis. The consistency of the two measurements was used to calculate the reliability of the WBB compared to the laboratory-grade force platform. However, the units of measurement for static balance ability by WBB and the laboratory-grade force platform were the same (millimetres).

### Instrument

The WBB was connected to a laptop (Intel Centrio, Windows 7, 5.2 GB RAM) via Bluetooth, with a sampling rate of 100 Hz. The MFU static balance test software was used to record the length of the path taken by the centre of gravity as calculated from data from four load sensors. Both the MFU software and the laboratory-grade force platform calculated the values for ‘total sway path’, ‘sway area’ and ‘sway velocity’. The ‘total sway path’ represents the Centre of pressure (COP) displacement (in mm) the centre of gravity travels within the 30 s period. Since there are several studies that use total sway path as the outcome measurement for comparison (7, 11), total sway path was selected as the variable of interest for this study.

### Statistical Analysis

Descriptive statistics were used to present the participant characteristics. The intraclass correlation coefficient (ICC_2,k_) was used to assess the agreement between the two measurement methods. The standard error of measurement (SEM) was calculated from SEM = SD x √(1-ICC) (12). The intraclass correlation coefficient (ICC_3,1_) and the Bland-Altman plot were then used to calculate test reliability. The results of the ICC can be interpreted as excellent for ICC of more than 0.75, moderate-to-good for those between 0.45 and 0.75 and poor for those less than 0.45 (12). One sample *t*-tests were used to determine the difference of the means between the two devices, and the mean difference between Trial 1 and Trial 2 for each condition. Where a significant difference was found, further analysis of the difference was not explored by Bland-Altman plots. Data analysis was performed using SPSS version 20.0 (SPSS Inc., Chicago, IL, USA) for Windows.

## Results

The baseline characteristics are presented in Table 1. In terms of validity as shown in Table 2, a poor agreement was found between the WBB and the laboratory-grade force platform while standing for all conditions, indicating that, at least in this study, the WBB is not valid as a clinical tool. ICC values of 0.03 were found for standing with the feet together with the eyes open [95%CI (−0.11 to 0.24)], 0.07 for standing with the feet together with the eyes closed (95%CI (−0.98 to 0.30)] and 0.04 for tandem standing with the eyes open [95%CI (−0.08 to 0.22)].

**Figure 2:**
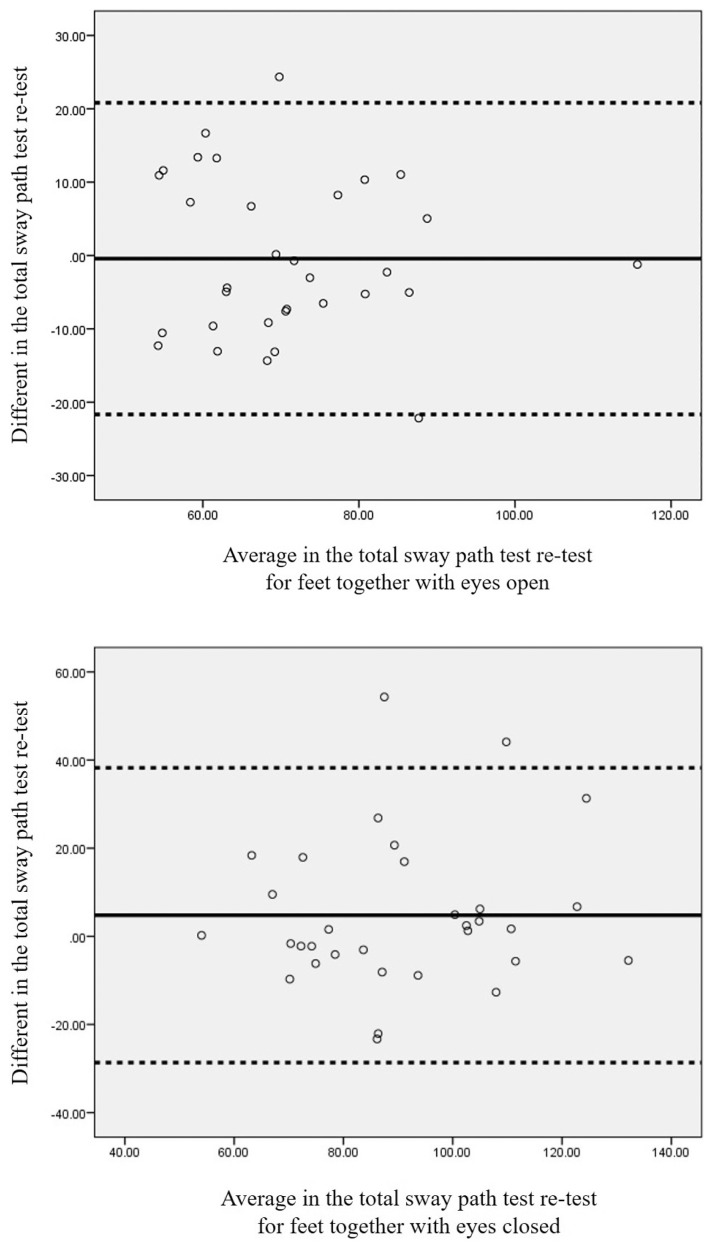

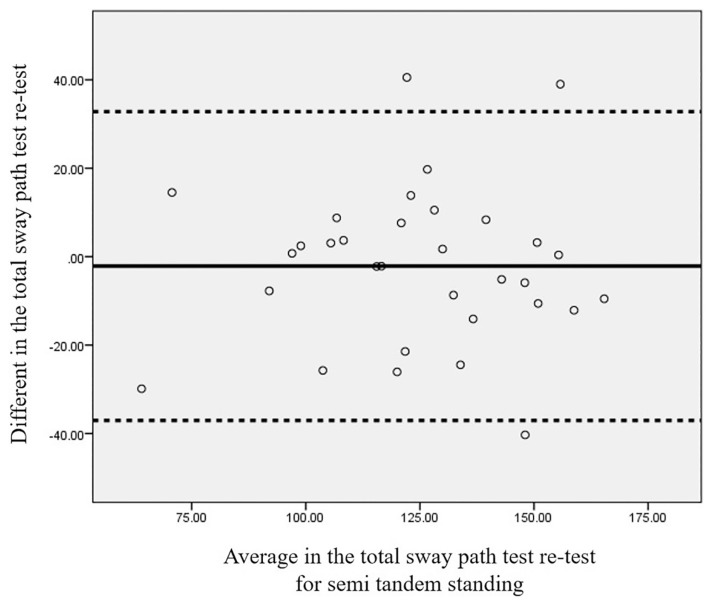
Bland-Altman plot representing the average of two trials for test-retest for the WBB

The consistency of the results presented in Table 3 shows that the ICC value has moderate to high reliability: 0.71 for standing with the feet together with the eyes open [95%CI (0.48 to 0.84)], 0.66 for standing with the feet together with the eyes closed (95%CI (0.42 to 0.82)) and 0.76 for tandem standing with the eyes open [95%CI (0.57 to 0.88)]. The Bland-Altman plot showed a negative bias of 0.42 mm for standing with the feet together with the eyes open (95% limits of agreement, from −21.66 mm to 20.81 mm; Figure 2), a positive bias of 4.78 mm for standing with the feet together with the eyes closed (95% limits of agreement, from −28.66 mm to 38.24 mm; Figure 2) and a negative bias of 2.12 mm for semi-tandem standing with the eyes open (95% limits of agreement, from −37.04 mm to 32.80 mm; Figure 2).

## Discussion

The purpose of this study was to investigate the validity and reliability of the WBB for balance assessment in healthy young adults. The results suggest the WBB has moderate to high reliability for this purpose, but poor validity.

There are several factors which may have affected the participants’ balance control. The visual cue given by the reference point on the wall could have been of assistance while standing (13), but this would be true for both platforms, and both eyes-open conditions. The eyes closed condition decreased the input from the visual system and increased the participants’ reliance on the somatosensory and vestibular systems. The more challenging balance task of tandem standing was used in this study (14).

The results suggest that, although reliable, the WBB had poor validity for balance assessment for all conditions in healthy young adults. This result is supported by Castelli et al., who also found low ICC values for inter-device reliability, but high ICC values for test-retest reliability between the WBB and the laboratory-grade force platform in healthy young adults (15). Clark et al., however, had different results, finding high ICC values when comparing the WBB and a laboratory-grade force platform (5). Notably, our protocol was different from that of Clark et al., who used a 15-s rest period between trials and a 60-s rest between devices.

The psychological experience might also have been a consideration in our study. The young adults might have had experience with game playing, and even with the WBB in particular. Consequently, they might have considered the WBB easy to manipulate while standing. The psychological process might have changed the neural activity in the brain, reflecting the familiarity with the activity (16).

In terms of reliability testing, although the ICC values suggested a moderate-to-high reliability in our study, the 95% confidence interval could be considered to be a wide range. Our results are supported by Clark et al., who also found a moderate-to-high reliability when testing young adults, although the research protocol was different (5). This is in contrast to Chang et al., who also investigated the reliability of the WBB in testing young adults (7). Notably, despite the different results, our protocol was the same as Chang et al. They found a low reliability in healthy young adults (7). However, although they employed the same resting periods of 10 min, their screening criteria for eligibility were different. There was, in addition, no data reported concerning the participants’ dynamic balance and lower extremity strength, which could have influenced results.

The correlation of the WBB with standard clinical tests for young adults, such as the Balance Error Scoring System (BESS), is unknown, which could be a limitation in our study. This gap in knowledge should be investigated in further studies. In addition, the WBB used with customised software in the ‘MFU static balance test’ will need further improvement due to its poor validity as a tool for balance assessment,

## Conclusions

The reliability of the WBB was found to be acceptable for clinical testing, but it was found to be invalid as a tool for balance assessment in healthy young adults. The WBB used with customised software in the ‘MFU static balance test’ will need further improvement for balance assessment.

## Figures and Tables

**Table 1:** Baseline Characteristics (*n* = 32)

Characteristics	Healthy Young Adults Mean (SD)
Gender a	
Male	8 (25.00)
Female	24 (75.00)
Age in years	19.06 (0.88)
Weight in kg	56.81 (13.75)
Height in m	1.61 (0.10)
Timed Up and Go test (s)	8.43 (1.11)
Five time sit to stand test (s)	6.98 (1.67)

a number (%)

**Table 2:** The agreement analysis of the total sway path by WBB and laboratory-grade force platform

	Wii Balance Board	Laboratory-grade Force Platform	Different (SD_diff_)^a^	ICC (95%CI)	SEM

	Mean (SD)				
Feet together with eyes open	70.85 (13.55)	178.62 (57.95)	107.77 (56.97)	0.03 (−0.11, 0.24)	55.91
Feet together with eyes closed	90.63 (19.11)	206.59 (55.18)	115.59 (52.62)	0.07 (−0.98, 0.30)	50.63
Semi tandem standing	124.67 (24.59)	502.87 (181.48)	378.19 (173.20)	0.04 (−0.08, 0.22)	169.70

a One sample *t*-test showed significant different between WBB and Laboratory-grade Force Platform (*P* < 0.01)

**Table 3:** Test re-test reliability of WBB

	Trial 1	Trial 2	Different (SD_diff_)	ICC (95%CI)	*P*-value^a^

	Mean (SD)				
Feet together with eyes open	70.64 (13.71)	71.06 (14.72)	−0.42 (10.83)	0.71 (0.48, 0.84)	< 0.01
Feet together with eyes closed	93.03 (21.76)	88.24 (20.05)	4.78 (17.06)	0.66 (0.42, 0.82)	< 0.01
Semi tandem standing	123.61 (26.36)	125.73 (25.95)	−2.12 (17.81)	0.76 (0.57, 0.88)	< 0.01

a intraclass correlation coefficient
